# A confirmatory bifactor analysis of the hospital anxiety and depression scale in an Italian community sample

**DOI:** 10.1186/1477-7525-12-84

**Published:** 2014-06-05

**Authors:** Luca Iani, Marco Lauriola, Massimo Costantini

**Affiliations:** 1 Department of Human Sciences, European University of Rome, Rome, Italy; 2 Department of Social and Developmental Psychology, University of Rome ‘Sapienza’, Rome, Italy; 3 Palliative Care Unit, Istituto di Ricovero e Cura a Carattere Scientifico (IRCCS) Arcispedale S. Maria Nuova, Reggio Emilia, Italy

**Keywords:** Measurement, Validity, Hospital anxiety and depression scale, Confirmatory factor analysis, Bifactor model

## Abstract

**Background:**

The Hospital Anxiety and Depression Scale (HADS) is a widely used self-report measure to assess emotional distress in clinical populations. As highlighted in recent review studies, the latent structure of the HADS is still an issue. The aim of this study was to analyze the factorial structure of the HADS in a large community sample in Italy, and to test the invariance of the best fitting model across age and gender groups.

**Methods:**

Data analyses were carried out on a sample of 1.599 participants proportionally stratified according to the Italian census population pyramid. Participants aged 18 to 85 years (females = 51.8%), living in eight different regions of Italy, voluntarily participated in the study. The survey questionnaire contained the HADS, Health Status questions, and sociodemographic variables.

**Results:**

Confirmatory factor analysis indicated that a bifactor model, with a general psychological distress factor and two orthogonal group factors with anxiety and depression, was the best fitting one compared to six alternative factor structures reported in the literature, with overall good fit indices [Non-normed Fit Index (NNFI) = .97; Comparative Fit Index (CFI) = .98; Root Mean-Square Error of Approximation (RMSEA) = .04]. Multi-group analyses supported total invariance of the HADS measurement model for males and females, and for younger (i.e., 18–44 years old) and older (i.e., 45–85 years old) participants. Our descriptive analyses showed that females reported significant higher anxiety and general distress mean scores than males. Moreover, older participants reported significant higher HADS, anxiety and depression scores than younger participants.

**Conclusions:**

The results of the present study confirmed that the HADS has good psychometric properties in an Italian community sample, and that the HADS scores, especially the general psychological distress one, can be reliably used for assessing age and gender differences. In keeping with the most recent factorial studies, our analysis supported the superior fit of a bifactor model. However, the high factor loadings on the general factor also recommend caution in the use of the two subscales as independent measures.

## Background

The Hospital Anxiety and Depression Scale (HADS) [1] is a popular self-report scale, originally developed to measure depression and anxiety among outpatients in nonpsychiatric hospital clinics (e.g., cancer, coronary heart disease). A number of studies have drawn attention to the positive qualities of the HADS, including brevity and easiness of use, good reliability and validity as well as efficiency in screening and case-finding [2-4].

Past reviews [2,5] also concluded that the HADS had a two-factor structure, more or less consistent with the original scoring rule. However, more recently, Cosco and colleagues [6] highlighted some contradictory results based on a comprehensive review of 50 studies, 24 of which based on Confirmatory Factor Analysis (CFA), that is the state-of-the-art methodology to evaluate the latent structure of psychometric scales. More specifically, while a three-factor model with correlated latent variables was reported as the best fitting one in 15 CFA studies, there were also seven studies supporting the validity of the two-factor model. In addition, analyses of clinical sample data were more likely to support the three-factor structure, although these factors were identified by different combinations of items. For instance, a number of studies tested Dunbar [7] model with Anhedonic Depression, Autonomic Anxiety, and Negative Affectivity [8,9]. Others tested Friedman [10] model [11,12] and Caci [13] model [8,14]; both models specified a single Depression factor, while the Anxiety factor was split into Psychic Anxiety and Psychomotor Agitation [10] or into Anxiety and Restlessness [13].

The HADS has been widely used as an effective tool to assess emotional distress in non-clinical populations [3]. Differently from clinical samples [8-10,15-18], CFA studies carried out on community or student samples not only were less frequent, but also the two-factor and three-factor models were about equally supported as best fitting ones [13,19]. For instance, Chan et al. [20] fitted a model with Anxiety and Depression very close to the original scoring rule, but with one depression item (i.e., #7) loading on anxiety see also [21]. By contrast, Martin and colleagues [22] provided support for a model similar to Dunbar’s, while others specified a different combination of items for the anxiety factors [13]. Notably, CFA studies that supported the two-factor model were carried out on large samples, i.e. more than 5.000 subjects [19,20].

Not surprisingly, given such a variety of factorial solutions, Norton et al. [23] carried out a meta confirmatory factor analysis study to systematically evaluate the HADS structure. Differently from the above cited studies, the authors concluded that a bifactor model (i.e., a model with a general factor affecting all items and two orthogonal group factors, accounting for a specific anxiety and depression variance, respectively) was the best one to account for the HADS structure in the majority of samples. Besides that, the bifactor model also provided an efficient way to model each item’s variance as the byproduct of general and specific unrelated components useful for applied purposes, thus becoming increasingly popular in clinical HADS research. In particular, an advantage in using bifactor model is the ability to decompose the variance of each item into one portion explained by the general factor and one portion explained by the group factor.

For instance, Luciano and colleagues [24] showed that the general factor (i.e., psychological distress) was positively associated with negative affect in fibromyalgia patients, while the specific depression factor was associated with low positive affect. Furthermore, the low factor loadings of anxiety items on their specific factor also cast doubts on the practical utility of HADS anxiety scores in that specific clinical population. Another study that tested the HADS bifactor model was recently carried out on patients with pain in different clinics in China [25]. This study showed that while the standard two-factor model showed a positive high correlation between anxiety and depression factors with pain severity, the bifactor model revealed a small negative correlation between specific depression and anxiety factors as well as a predictive association of pain severity only with the general distress factor. Both studies suggested that, under specific clinical circumstances, the interpretation of specific HADS scores was unwarranted and that the HADS could be a valid measure of general distress.

In summary, since the factorial structure of the HADS is still an issue, especially for what concerns the non-clinical population, the present study aims to analyze the factorial structure of the HADS in a large community sample in Italy. A test of the bifactor model not involving clinical samples is a very recent trend in HADS research and, to the best of our knowledge, published studies are still rare [26]. Hence, our study aims to cross-validate previous findings supporting the superior performance of the bifactor model over other alternative HADS models. As a byproduct of our extensive data collection, we also report the psychometric properties and descriptive statistics for an Italian community sample of different age and gender groups.

## Methods

### Participants

A total of 1.599 participants aged 18 to 85 years (median age category 45–54 years; females = 51.8%), living in eight different regions of Italy, voluntarily participated in a study presented as a survey of citizen satisfaction in Italy. Consistent with the Italian population pyramid, age and gender quotas were imposed to collect data from each region see also [27]. The sample’s characteristics are reported in Table 1.

**Table 1:** Sociodemographic characteristics of the sample

	**Males**		**Females**		**Total**	
	***N***	**%**	***N***	**%**	***N***	**%**
**Age category**						
18-24	65	8.44	70	8.4	135	8.4
25-34	120	15.58	118	14.2	238	14.9
35-44	160	20.78	152	18.3	312	19.5
45-54	145	18.83	144	17.4	289	18.1
55-64	120	15.58	124	15.0	244	15.3
≥ 65	160	20.78	221	26.7	381	23.8
**Civil status**						
Married	365	47.8	369	44.6	764	48.0
Not married	399	52.2	458	55.4	857	52.0
**Education (years)**						
5	47	6.2	94	11.4	141	8.9
6-8	161	21.3	164	20.0	325	20.6
9-13	363	48.0	341	41.5	704	44.6
≥ 14	186	24.6	223	27.1	409	25.9
**Level of chronic disease/limitation**						
No disease/no limitation	578	75.06	576	69.48	1154	72.17
Disease/no limitation	56	7.27	67	8.08	123	7.69
Disease/some limitation	102	13.25	153	18.46	255	15.95
Disease/severe limitation	34	4.42	33	3.98	67	4.19
**Economic status**						
Below the poverty line	463	61.16	499	60.85	962	61
Above the poverty line	294	38.84	321	39.15	615	39

Trained interviewers recruited potential participants from public places (e.g., streets), waiting places (e.g., railway stations) or from places open to the public (e.g., senior centers). Before obtaining informed consent, participants were given information about the study aims and characteristics. Less than 5% of the subjects refused to participate to the survey.

### Measures

Besides the Italian HADS translation, already used for the clinical sample [28], gender, age, marital status, employment status, education level and size of the municipality of residence were collected.

#### Health status

Two questions taken from the Italian “Multipurpose survey on households: aspects of daily life – general part” included in the National Statistic Programme [29], were used to survey participants’ health status. In particular, we asked “Do you suffer from a chronic disease or health problems?” (*yes*/*no*), and “Have you been limited in activities which people normally carry out due to a health problem lasting at least six months?” (*no limitation*/*some limitations*/*severe limitations*). These questions were combined to create a single health status index, as follows: No chronic disease/chronic disease and no limitation/chronic disease and some limitations/chronic disease and severe limitations.

#### Economic status

Research participants were also asked to provide an accurate estimate of their average consumption expenditure level as well as to indicate the number of residents in their household. We used this information as an indicator of participants’ relative poverty, according to the International Standard of Poverty Line [30]. Accordingly, participants were classified as relatively poor or relatively rich if they were below or above the poverty line, respectively.

### Data analyses

#### Confirmatory factor analysis

The following first-order factor models were tested: the Razavi [31] model with a single one order factor; the Zigmond-Snaith [1] model with odds and even items for anxiety and depression, respectively; the Moorey [21] model with Anxiety (Items 1, 3, 5, 9, 11, and 13) and Depression (Items 2, 4, 6, 7, 8, 10, 12, and 14); the Dunbar [7] model with Anhedonic Depression (Items 2, 4, 6, 8, 10, 12, and 14), Autonomic Anxiety (Items 3, 9, and 13), and Negative Affectivity (Items 1, 5, 7, and 11); the Friedman [10] model with Depression (Items 2, 4, 6, 8, 10, 12, and 14), Psychic Anxiety (Items 3, 5, 9, and 13) and Psychomotor Agitation (Items 1, 7, and 11); the Caci [13] model with Depression (Items 2, 4, 6, 8, 10, 12, and 14), Anxiety (Items 1, 3, 5, 9, and 13) and Restlessness (Items 7, 11, and 14). In addition, we tested a bifactor model, with a general factor and two group factors with Anxiety (Items 1, 3, 5, 7, 9, 11, and 13) and Depression (Items 2, 4, 6, 8, 10, 12, and 14).

Since the data were not normally distributed (Mardia’s normalized coefficient = 41.68), maximum likelihood (ML) robust estimators were used. Accordingly, we reported fit statistics based on the Satorra–Bentler scaled chi square (SBχ^2^) as available in EQS 6.2 [32]. Because of the large sample size, we expected all models to have a significant chi-square value. Therefore, more “practical” indices of fit were used to evaluate each model’s fit as well as to compare alternative models, according to the recommended cut-offs [33,34]. More specifically, a chi-square to degree of freedom ratio value is used to minimise the impact of sample size on the model chi-square; values less than 2 indicate good fit. The Akaike Information Criterion (AIC) is a statistic generally used to compare the fit of non-nested or non-hierarchical models; lower values indicate a better fitting model. Both the comparative fit index (CFI) and the non-normed fit index (NNFI) result from a comparison between the hypothesized model’s chi square with the independence model’s one. Values greater than .95 are recommended for both indices. The root mean squared error of approximation (RMSEA) is instead a ‘badness of fit’ index assessing the difference between the reproduced covariance matrix and the population covariance matrix. RMSEA very close to 0 indicate almost perfect fit; values less than .05 are recommended as they reflect a small approximation error. The 90% confidence interval (CI) around the RMSEA point estimate is also commonly reported to indicate the possibility of close or exact fit.

Some physical and mental health outcomes (i.e. depression) show a different trend according to variation in age and gender, namely older adults frequently score higher in depression [35]. Therefore, we tested the invariance of the best-fitting model across different gender and age groups. A first multi-group analysis was based on two groups comprised of 770 males and 829 females, respectively. Then, a second multigroup analysis was based on two age groups composed of 685 and 914 participants aged under and over 45 years, respectively.

## Results

### Descriptive statistics

The general distress mean score reported for the whole sample was 13.0 (*SD* = 7.8), whereas the anxiety and depression scores were 7.6 (*SD* = 4.4) and 5.4 (*SD* = 4.0), respectively. Cronbach’s alpha reliability coefficients were fairly high (.86, .80, and .89 for anxiety, depression and general distress scores, respectively). Next, we compared the Anxiety, Depression and General Distress average scores resulting from this study with the average scores resulting from similar studies carried out on community samples [13,20,36-38]. The mean scores assessed in our study were slightly higher than those reported in the literature. For instance, Anxiety in our sample was above 7.3, that is the maximum average score found in the literature [13]. Likewise, Depression in our sample was as large as 5.4, also reported by Chan et al. [20]. Last, the General Distress was above 12.3, reported by Chan and colleagues [20]. Table 2 reports means and standard deviations for HADS scores broken down by gender and age.

**Table 2:** HADS mean scores

	**Males**						**Females**						**Whole sample**					
	**Anxiety**		**Depression**		**Total**		**Anxiety**		**Depression**		**Total**		**Anxiety**		**Depression**		**Total**	
	***M***	***SD***	***M***	***SD***	***M***	***SD***	***M***	***SD***	***M***	***SD***	***M***	***SD***	***M***	***SD***	***M***	***SD***	***M***	***SD***
18-24 y	5.0	3.3	3.5	2.7	8.5	5.2	6.4	3.8	3.7	2.9	9.9	5.7	5.7	3.6	3.6	2.8	9.2	5.4
25-34 y	6.7	4.1	4.4	3.5	11.2	7.1	7.2	3.9	4.0	3.0	11.2	6.4	7.0	4.0	4.3	3.3	11.2	6.7
35-44 y	6.9	5.0	5.0	4.4	11.9	8.8	8.1	3.7	5.3	3.8	13.4	6.8	7.5	4.4	5.1	4.1	12.6	8.0
45-54 y	7.2	4.3	5.5	4.3	12.8	8.1	8.5	4.5	5.4	3.4	13.9	7.1	7.9	4.5	5.5	3.9	13.3	7.7
55-64 y	7.5	4.7	6.8	4.6	14.4	8.7	8.6	4.6	6.3	4.5	14.9	8.6	8.1	4.7	6.6	4.5	14.7	8.6
≥ 65 y	7.7	4.4	6.4	4.2	14.1	7.9	8.3	4.6	6.3	4.2	14.7	8.0	8.0	4.5	6.3	4.1	14.4	7.9
all	7.0	4.5	5.5	4.2	12.5	8.1	8.0	4.3	5.4	3.9	13.4	7.5	7.6	4.4	5.4	4.0	13.0	7.8

The analysis of variance revealed significant gender differences in Anxiety (*F*_1,1585_ = 19.85, *p* < .001) and in the total HADS scores (*F*_1,1575_ = 5.70, *p* = .017), but not in Depression. As we compared young adults participants (18–44 years old) with older adults (45–85 years old), we found significant age differences in anxiety (*F*_1,1585_ = 21.66, *p* < .001), depression (*F*_1,1584_ = 62.76, *p* < .001), and general distress scores (*F*_1,1575_ = 46.23, *p* < .001). The analysis of variance of HADS scores by other sample’s characteristics showed that participants with an upper level of education (i.e., ≥ 14 years) reported significantly lower anxiety, depression and general distress scores than those with a lower level (i.e., five years, and six to eight years). Moreover, relatively poor participants scored significantly higher than relatively rich ones in all three HADS scores (*p* < .01). As expected, participants with a worse health status (i.e., disease with severe limitations) reported significantly higher scores than participants with a higher health status in all three HADS scores. No differences were found in all three HADS scores between married and not married participants.

### Confirmatory factor analysis

Table 3 reports absolute and relative fit indices for single order factor models as well as for Norton’s bifactor model (see Methods).

**Table 3:** Fit statistics for the proposed models of the HADS

	**χ**^**2**^	***df***	***p***	**SB χ**^**2**^	***p***	**χ**^**2**^**/df**	**SB χ**^**2**^**/df**	*AIC	*CFI	*NNFI	*RMSEA (90% CI)
1. Razavi et al. (1990) [30]	1259.54	91	<.001	1052.04	<.001	13.84	11.56	896.04	.84	.81	.09 (.08-.09)
2. Zigmond & Snaith (1983) [1]	476.03	76	<.001	397.35	<.001	6.26	5.23	245.35	.95	.94	.05 (.05-.06)
3. Moorey et al. (1991) [21]	447.93	76	<.001	373.17	<.001	5.89	4.91	221.17	.95	.94	.05 (.05-.06)
4. Dunbar et al. (2000) [7]	472.92	74	<.001	393.72	<.001	6.39	5.32	245.72	.95	.94	.05 (.05-.06)
5. Friedman et al. (2001) [10]	462.77	74	<.001	386.23	<.001	6.25	5.22	238.23	.95	.94	.05 (.05-.06)
6. Caci et al. (2003) [13]	471.71	74	<.001	394.36	<.001	6.37	5.33	246.36	.95	.94	.05 (.05-.06)
7. Bifactor	238.53	63	<.001	200.21	<.001	3.79	3.18	74.21	.98	.97	.04 (.03-.04)

*Note*: SBχ^2^ = Satorra-Bentler chi-square; *= robust statistics.

Recommendations and cut-off points for model fit [33,34]: Low χ^2^ relative to degrees of freedom with an insignificant *p* value (*p* > 0.05); relative/normed chi-square (χ^2^/df) smaller than .05; smaller values of AIC; CFI and NNFI equal to or greater than .95; RMSEA equal to or less than .08; 90% confidence intervals for the RMSEA close to RMSEA.

**Figure 1:**
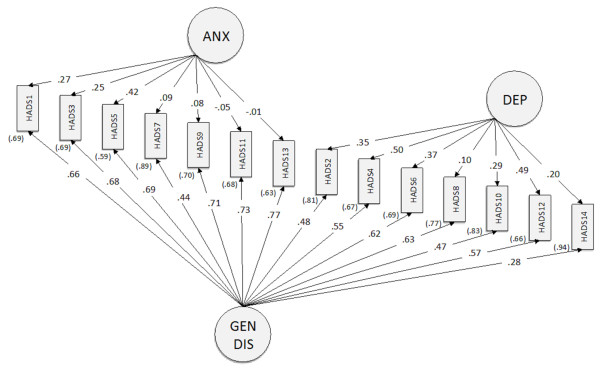
**The hospital anxiety and depression scale bifactor structure.** The standardized factor loadings, with measurement error terms in parenthesis, are reported. ANX = Anxiety; DEP = Depression; GEN DIS = General Distress.

As expected, the chi-square statistics were statistically significant for all models due to the large sample size. The single-factor model [31] provided a poor fit to the data, and was indeed rejected. By contrast, all two-factor and three-factor models [1,7,10,13,21] had acceptable fit indices, except for the NNFI that only approached the acceptability threshold. Finally, we tested the bifactor model that outperformed all the other models tested in this study. Figure 1 reports the Hospital Anxiety and Depression Scale bifactor structure.

A first multi-group analysis was based on two groups comprised of 770 males and 829 females, respectively. A second multi-group analysis was based on two age groups: 685 participants aged 18–44 years, and 914 participants aged 45–85 years. The multi-group analysis by gender supported the configural invariance of the bifactor model (SBχ^2^ = 271.22; *df* = 126; CFI = .98; NNFI = .97; RMSEA = .04; 95% C.I. [.03–.04]), pointing that the same number of factors and factor-indicator correspondence were equivalent across both gender groups. Fit statistics were used as a baseline onto which factor loadings and measurement error invariance were compared. Subsequently, a more stringent type of invariance was evaluated by considering a model with equality constraints imposed to factor loadings. The imposition of equality constraints on all factor loadings fitted the data well (SBχ^2^ = 270.21; *df* = 140; CFI = .98; NNFI = .97; RMSEA = .03; 95% C.I. [.03–.04]), pointing that the same unstandardized factor loadings of each indicator were equivalent across groups. There was no statistically significant scaled chi-square difference between the restricted model and the configural invariance model. A subsequent model with equality constraints imposed to both factor loadings and error variances also resulted in a good fit (SBχ^2^ = 264.96; *df* = 154; CFI = .98; NNFI = .98; RMSEA = .03; 95% C.I. [.02–.03]), with a nonsignificant scaled chi-square difference relative to the factor loadings invariance model.

Finally, a second multi-group analysis with different age groups was carried out in order to test the invariance by age. The multi-group analysis by age showed that the same number of factors and factor-loading pattern were equivalent across both age groups, supporting the configural invariance of the bifactor model (SBχ^2^ = 256.10; *df* = 126; CFI = .97; NNFI = .96; RMSEA = .04; 95% C.I. [.03–.05]) and its fit statistics were used as a baseline onto which more stringent models were compared. A model with equality constraints imposed to factor loadings also fitted the data well (SBχ^2^ = 255.83; *df* = 140; CFI = .97; NNFI = .97; RMSEA = .03; 95% C.I. [.03–.04]), and no statistically significant scaled chi-square difference between the restricted model and the configural invariance model was found. The imposition of equality constraints on both factor loadings and error variance fitted the data well (SBχ^2^ = 247.55; *df* = 154; CFI = .98; NNFI = .97; RMSEA = .03; 95% C.I. [.02–.04]), and there was no statistically significant scaled chi-square difference between the more restricted model and the factor loadings invariance model.

## Discussion

The HADS is a brief and widely used measure of anxiety and depression. Not surprisingly, its factor structure has been extensively studied during the past two decades. Earlier studies showed that a two-factor solution emerged in exploratory analyses of the HADS [1,21]. However, during the past decade CFA has gained popularity as the standard method for assessing the construct validity of psychometric scales supported by previous research or largely driven by theory, like the HADS. Differently from EFA, CFA provides a more thorough and theoretically driven test of psychometric assumptions in that it requires *a priori* specification for the statistical model representing item-factor relations. While a detailed coverage of this topic is beyond the scope of the paper, suffice it to say that CFA requires to specify: the number of factors and whether or not the factors in the model are correlated, which variables are expected to load onto which factor(s), which model’s parameters (e.g., factor loadings, error terms variance) should be “freely estimated” or “fixed” to a specific value. More importantly, as a result of each of these methodological choices, CFA provides a measure of model’s fit, that can be either accepted or rejected [39].

More recent studies, based on CFA, pointed out a variety of solutions, ranging from one to three factors, each loading different combination of items [7,10,13,31]. In addition, studies including non-clinical samples were less frequent, although the HADS is deemed a valid psychological distress measure in community samples [26,36]. Recent developments in modeling the HADS structure [6,24] suggested that a bifactor model could fit HADS data better than any single-order or hierarchical factor. Moreover, the bifactor model can decompose the variance of each item into one portion explained by the general distress factor, and one portion explained by depression or anxiety group factors, thereby providing an insight on the appropriateness of factor scores to assess general distress, anxiety and depression in community samples.

Thus, a test of the bifactor model not involving clinical samples is becoming a very recent trend in HADS research, although published studies are still rare for community samples [26]. Therefore, we collected data from a relatively large Italian community sample and tested single-order and bifactor models to expand on the above cited HADS literature. Our results were overall consistent with the meta-analytic work by Cosco et al. [6], indicating that a bifactor model with a general factor affecting all items and two orthogonal group factors, accounting for a specific anxiety and depression variance, was the best fitting one compared to six alternative factor structures reported in the literature [1,7,10,13,21,31].

Besides that, the inspection of model’s parameters lead us to conclude that in our community sample, as in similar studies testing bifactor models and involving both clinical and community participants, all HADS items, except item #14, loaded more highly on the general factor than on each specific group factor. As recommended by Cosco et al. [6], findings like ours should induce caution in the interpretation of group factors scores, posing the clinical usefulness of the two subscales as questionable. On the other hand, they support the suitability of a reliable and valid single measure of general psychological distress.

In our analysis, the factor loadings on the general psychological distress factor were > .40 for all HADS items except for #14. By contrast, only one Anxiety item (#5) and two Depression items (#4, #12) had factor loadings > .40 on their respective group factor. Thus, our results indicated that scoring the general distress factor is more appropriate for screening community as well as clinical populations [23,26], also in the light of evidence that Anxiety and Depression are usually comorbid and often yield highly correlated assessments [40,41]. Our findings are also consistent with a previous HADS Italian validation study that suggests the presence of a common area of emotional disturbance, shared by both factors, to interpret the overlap between Anxiety and Depression [28].

As a byproduct of our data collection, we also studied age and gender differences in HADS scores. Before doing that, we addressed the issue of measurement invariance that is critical in order to appraise between-group differences as meaningful [42]. In fact, metric invariance ensures that HADS items had the same meaning for males and females or for younger and older adults. Our analysis demonstrated the total invariance across age and gender, and therefore supported the HADS use for assessing and comparing specific subgroups on the same standard.

Thus, our descriptive analyses showed that female participants reported statistically significant higher anxiety mean scores than males. This result is consistent with most previous HADS studies on community samples [13,20,26,36,37]. Female participants reported statistically significant higher general distress mean scores than males, but this result is consistent with fewer studies [13,36]. No differences have been found in depression scores between males and females. Although this result is consistent with previous studies [13,37,38], other scholars have found different results [20,26,36]. As expected, older participants reported significant higher HADS, anxiety and depression scores than younger participants. Other studies [19,26,43] reported similar results only for depression mean scores.

Overall, our findings are consistent with recent trends in the HADS psychometric literature as they indicated that the bifactor model outperformed other popular accounts of the HADS factor structure. However, it is worth noting some limitations to our conclusions. The most relevant was that our sampling method, although based on proportional stratified sampling by age and gender according to the national census, was non-probabilistic. So, our estimates of average scores could be to some extent biased by interviewers, who recruited participants from public places, waiting places or from places open to the public. Nevertheless, other studies assessing HADS factor structure [20,36] used convenience samples.

Despite these limitations, the results of the present study confirmed that the HADS has good psychometric properties in an Italian community sample as well as in the Italian clinical study [28], and that the HADS scores, especially the general psychological distress one, can be reliably used for assessing age and gender differences. In keeping with the most recent factorial studies, our analysis supported the superior fit of a bifactor model with a general psychological distress factor and two group factors with anxiety and depression.

## Conclusions

The HADS has been shown to be a reliable and valid measure to detect psychological distress in an Italian community sample. However, the high factor loadings on the general factor also recommend caution in the use of the anxiety and depression subscales as independent measures.

## Abbreviations

HADS: The hospital anxiety and depression scale; CFA: Confirmatory factor analysis; ML: Maximum likelihood; SBχ^2^: Satorra–Bentler scaled chi square; AIC: Akaike Information Criterion; CFI: Comparative Fit Index; NNFI: Non-normed Fit Index; RMSEA: Root mean square error of approximation; CI: Confidence interval.

## Competing interests

The authors declare that they have no competing interests.

## Authors’ contributions

LI, ML and MC discussed the contents of this article together. LI and ML developed the theoretical framework, elaborated the research hypotheses, devised the methodological content, and analyzed the data. MC provided a significant contribution to the theoretical framework and manuscript revision. The final version of the manuscript was written by LI and ML. All authors read and approved the final manuscript.
